# Dual vs. single plating in distal femoral fractures: a systematic review and meta-analysis

**DOI:** 10.1186/s13018-025-06309-7

**Published:** 2025-10-24

**Authors:** Ahmed Oun, Hamdy Khaled Sabra, Omar Abdelaziz, Islam Saeed Elhois, Ahmad Omar Saleh, Khaled Hemdan, Ahmed O. Sabry

**Affiliations:** 1 Faculty of Medicine, Tanta University Hospital, Tanta University, Tanta, Egypt; 2 Faculty of Medicine, Alexandria University, Alexandria, Egypt; 3 Department of Orthopedics, Qena General Hospital, Ministry of Health and Population, Qena, Egypt; 4 The University of Jordan, Amman, Jordan; 5 Faculty of Medicine, October 6 University Hospital, October 6 University, Giza, Egypt; 6 Orthopedic Department, Cairo University, Cairo, Egypt

## Abstract

**Background:**

Distal femoral fractures, comprising 0.4% of all fractures, present significant management challenges due to their complex anatomy and associated complications. While single plating (SP) is commonly used, its biomechanical limitations in complex fractures have prompted interest in dual plating (DP), which may offer superior stability. This systematic review and meta-analysis aimed to compare clinical outcomes between single and dual plating techniques in distal femoral fractures.

**Methods:**

A comprehensive literature search was conducted in PubMed, Scopus, and Web of Science up to May 2025, following PRISMA guidelines. Thirteen retrospective studies comparing dual vs. single plating in distal femoral fractures (DFF) involving 1,015 patients (654 SP, 361 DP) met inclusion criteria. Outcomes assessed included union rates, operation time, blood loss, postoperative range of motion (ROM), functional scores, complications, and reoperation rates. Data were analyzed using random-effects models, with heterogeneity assessed via I^2^ statistics.

**Results:**

DP was associated with significantly higher union rates with odds of union approximately five times greater than SP (OR = 5.34, 95% CI: 2.23–12.79; *p* = 0.0002), shorter union times (MD= -3.08, 95% CI: -5.18, -0.99; *p* = 0.004), a 73% lower odds of nonunion (OR = 0.27, 95% CI: 0.14, 0.53; *p* = 0.0002), an 89% lower odds of malunion (OR = 0.11, 95% CI: 0.02, 0.54; *p* = 0.007), and an 84% lower odds of delayed union (OR = 0.16, 95% CI: 0.04, 0.68; *p* = 0.01). However, DP resulted in longer operative times (MD = 27.19, 95% CI: 23.11–31.28; *p* < 0.00001). SP demonstrated superior postoperative knee ROM (*p* = 0.02). No significant differences were observed between groups in hospital stay, reoperation rates, superficial infections, or overall complications; *p* < 0.05. Heterogeneity was low for most outcomes, except for blood loss (I^2^ = 84%), flexion contracture (I^2^ = 89%), and union time (I^2^ = 69%), reflecting variability in surgical technique and case complexity.

**Conclusions:**

Dual plating offers superior fracture healing without increasing complication rates, making it preferable for DFF. Single plating may remain advantageous for patients prioritizing postoperative knee mobility.

**Supplementary Information:**

The online version contains supplementary material available at 10.1186/s13018-025-06309-7.

## Introduction

Distal femoral fractures, accounting for approximately 0.4% of all fractures and 3% of femoral fractures, are severe injuries often resulting from high-energy trauma such as motor vehicle accidents or falls [1, 2]. These fractures involve the metaphyseal and condylar regions of the distal femur, just above the knee joint [3].

Due to their proximity to the knee joint and the complex anatomy of the distal femur, these fractures can lead to significant morbidity, including joint stiffness, malunion, nonunion, and post-traumatic arthritis [4]. The optimal surgical management of distal femoral fractures remains a subject of ongoing debate, particularly concerning the choice between single and dual plating techniques.

Single plating, typically involving a lateral locking plate, has been a common approach for stabilizing distal femoral fractures. This technique offers advantages such as less soft tissue dissection and a relatively straightforward surgical procedure [5].

However, for complex intra-articular fractures or those with significant metaphyseal comminution, single lateral plating may not provide sufficient biomechanical stability, potentially leading to higher rates of complications such as nonunion or implant failure [6, 7].

In contrast, dual plating, which involves the application of both medial and lateral plates, is often considered for more complex distal femoral fractures. Biomechanical studies have demonstrated that dual plating provides superior axial and torsional stiffness compared to single lateral plating, offering enhanced stability for fracture healing [8, 9].

This increased stability is particularly beneficial in cases with metaphyseal comminution or osteoporotic bone, where robust fixation is crucial [10]. Despite its biomechanical advantages, concerns exist regarding dual plating, including increased soft tissue dissection, potential for greater blood loss, and a higher risk of infection [11].

The existing literature presents conflicting evidence regarding the superiority of dual versus single plating in distal femoral fractures. Some studies suggest that dual plating leads to improved union rates and better functional outcomes, particularly in comminuted fracture patterns [11, 12]. Conversely, other research indicates no significant differences in nonunion rates, blood loss, or functional outcomes between the two approaches [13]. In particular, while certain reports highlight faster or more reliable healing with dual plating, others fail to demonstrate advantages in postoperative range of motion, underscoring the uncertainty surrounding its overall clinical value. Therefore, a comprehensive systematic review and meta-analysis is warranted to clarify the comparative efficacy and safety of dual versus single plating in the treatment of distal femoral fractures.

## Methods

A comprehensive literature search was conducted for studies published up to May 2025, utilizing PubMed, Scopus and Web of Science databases to retrieve studies comparing single plating vs. dual plating in distal femoral fractures.

The search strategy employed the following combination of Medical Subject Headings (MeSH) terms and using the following key word and its synonymous: distal femoral fracture, dual plate, single plate, medial, and lateral plate (detailed search strategy in supplementary) .This systematic review adhered to the Preferred Reporting Items for Systematic Reviewers and Meta-Analyses (PRISMA) guidelines (Supplementary file 1).

Inclusion criteria encompassed: (i) all patients were adults (>18 years old) diagnosed with a fracture of the distal femur; (ii) the intervention was a double plate surgical treatment vs. single plate; (iii) at least two indicators should be included: operation time, intraoperative blood loss, fracture healing time, range of motion (ROM) of the knee joint post-operation, overall excellent and good rates of Schatzker–Lambert functional score (20), and postoperative complications; (iv) randomized controlled trials and cohort studies; (v) the follow-up time was at least 6 months.

Exclusion criteria comprised (i) non-English language articles, review articles, case report, letter, or conference abstract (ii) research without access to the original text or extracted data; (iii) cadaver studies and sawbones studies and synthetic femur.

Four reviewers extracted relevant information from each included study, with subsequent verification by two independent reviewers to ensure accuracy.

Extracted data included year of publication, country of origin, study design, sample size, patient demographics, surgical technique (single plating or dual plating), and outcomes (Union time, operation time, blood loss, length of hospital stay, follow up, Knee Society Score, Modified RUST Score, union rate, nonunion rate, delayed union rate, malunion rate, reoperation rate, knee range of motion (ROM), knee flexion contracture, superficial surgical site infection (SSI), Knee joint stiffness rate).

The Newcastle–Ottawa Scale (NOS) was used to assess the quality of the included studies [14]. This tool evaluates three main domains: selection of study groups, comparability of groups, and outcome assessment.

Each study was scored based on these criteria, with higher scores indicating better quality. Two independent reviewers conducted the quality assessment, and any discrepancies were resolved through discussion. The detailed quality assessment results are provided in Table 4.

Mean difference (MD) with 95% Confidence Intervals (CIs) were pooled for continuous outcomes, and odds ratio (OR) for dichotomous outcomes. with OR >1 indicating greater odds of the event occurring in the single plating group compared with the dual plating group. Odds ratios were the primary effect measure. In addition, absolute risk differences (RDs) were computed to complement relative measures. A p-value < 0.05 was considered statistically significant for overall effect estimates.

Publication bias was assessed and a funnel plot was provided. Egger’s regression test using the standard error of the observed outcomes was applied to check for funnel plot asymmetry.

The DerSimonian and Laird random-effects model was applied when the heterogeneity can’t be resolved by fixed effect model. Heterogeneity was assessed using the Cochran Q test and I^2^ statistics, with *p* < 0.10 and I^2^ < 25% considered indicative of low/non-significant heterogeneity, following Cochrane’s guidelines. Statistical analyses were performed using Review Manager (RevMan) Version 5.4.1 (The Cochrane Collaboration, Copenhagen, Denmark).

## Results

**Fig. 1:**
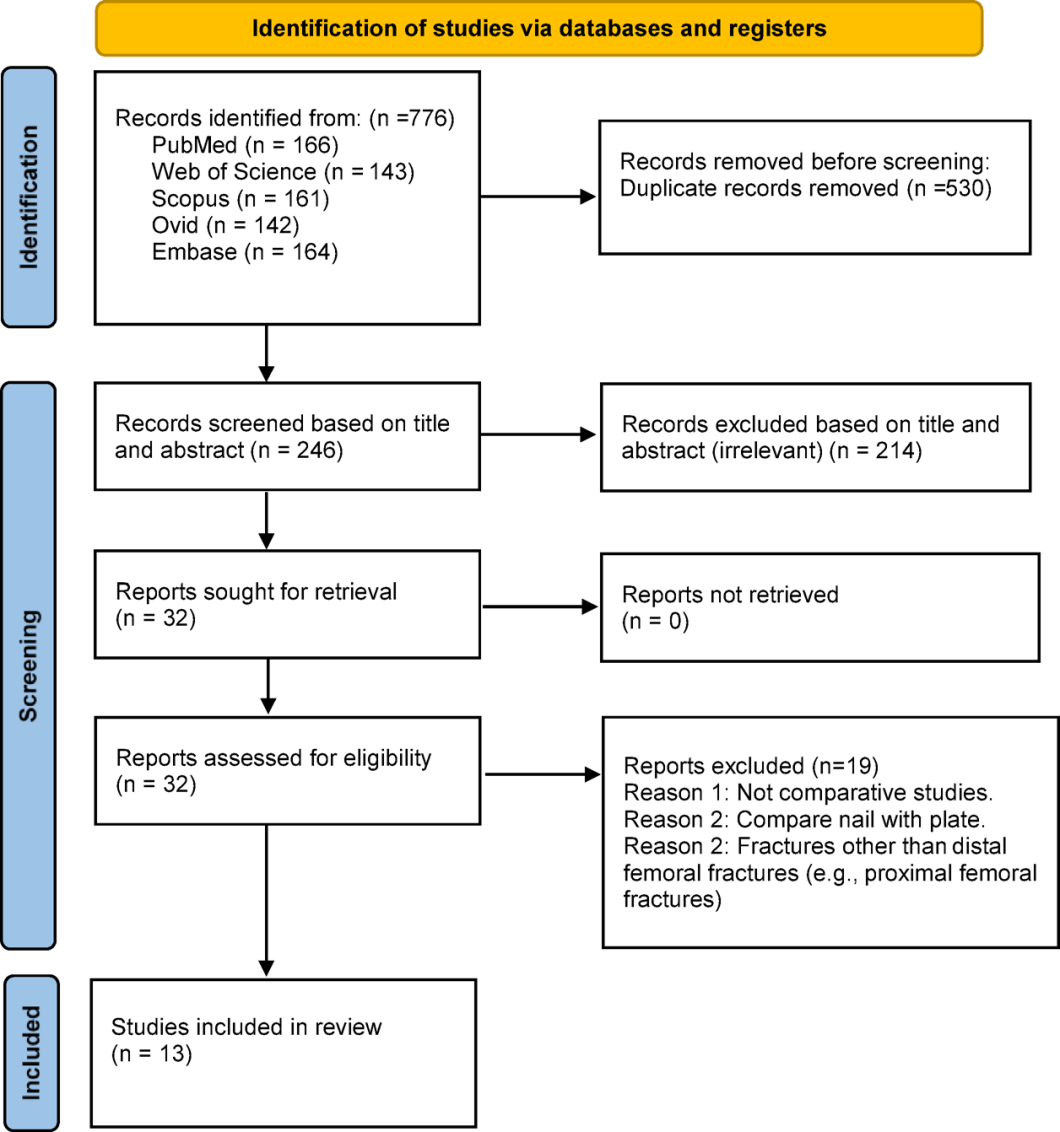
PRISMA flow diagram depicting the selection of the included articles

Initially, a total of 776 articles were identified. After the removal of 530 studies as duplicates, 246 articles were subjected to screening based on their titles and abstracts to assess eligibility. Of these studies, 32 articles were deemed suitable for full-text review. Upon full text screening, 19 studies were excluded due to reasons including non-English language, and lack of information on dual vs. single plating, fractures other than distal femur fractures and review articles. Ultimately, 13 articles were included in this review. Figure 1 illustrates the entire study selection process.

The methodological quality of the included studies was assessed using the Newcastle–Ottawa Scale (NOS) for cohort studies. All of the studies show high quality.

About the Selection domain, all the studies demonstrate high quality in representativeness of the exposed cohort, selection of the non-exposed cohort, ascertainment of exposure, and demonstration that the outcome of interest was not present at the start of the study.

About Comparability, all studies showed statistically comparable results at baseline, and appropriate tests were used.

Regarding the outcome, all studies showed an independent blind assessment, the follow-up was adequate, sufficient, and accounted (Table 1a, b).

**Table 1:** Clinical outcomes of single vs. dual plating in distal femoral fractures

a										
Study ID	Groups	Operation time (min)	Blood loss (ml)	Time to union (weeks)	Length of stay (days)	Knee Society Score	Modified RUST score	Time to full weight bearing	Knee ROM	Flexion contracture
Incheol Kook	SP (*n* = 50)	131 (33.6)	427.5 (144.5)	22.3 (4.1)	NA	NA	NA	NA	119.5 (8.3)	NA
	DP (*n* = 27)	143.7 (38.1)	444.7 (144.5)	16.7 (3.8)	NA	NA	NA	NA	113.9 (14)	NA
Makoa Mau	SP (*n* = 229)	NA	NA	NA	9 (9)	NA	NA	NA	NA	NA
	DP (*n* = 70)	NA	NA	NA	9 (10)	NA	NA	NA	NA	NA
Jerrod Steimle	SP (*n* = 91)	162.5 (61.54)	186 (257)	29.5 (10.65)	NA	NA	NA	13.5 (6.88)	NA	NA
	DP (*n* = 5)	194.7 (12.09)	150 (33.5)	22.1 (10.65)	NA	NA	NA	8.4 (6.88)	NA	NA
Tyler Thorne	SP (*n* = 133)	154 (74.95)	NA	NA	5 (3)	NA	NA	NA	NA	NA
	DP (*n* = 37)	190 (89.46)	NA	NA	5.67 (3.08)	NA	NA	NA	NA	NA
P. Kriechling	SP (*n* = 66)	NA	NA	NA	12 (6.06)	NA	NA	NA	NA	NA
	DP (*n* = 15)	NA	NA	NA	12 (9)	NA	NA	NA	NA	NA
Murat Çalbıyık	SP (*n* = 29)	NA	NA	14.9 (2.1)	7 (1.3)	84.3 (7.2)	11 (2.4)	NA	112 (11)	4 (2.77)
	DP (*n* = 27)	NA	NA	13.5 (2.6)	7 (1.3)	83.5 (5.5)	12 (1.6)	NA	108 (9.4)	NA
Nicholas A. Andring	SP (*n* = 34)	NA	NA	NA	NA	NA	NA	NA	120 (27.5)	NA
	DP (*n* = 38)	NA	NA	NA	NA	NA	NA	NA	111 (13.2)	NA
Chang Heng Liu	SP (*n* = 15)	NA	NA	141.21 (224.01)	NA	NA	NA	NA	NA	NA
	DP (*n* = 39)	NA	NA	188.66 (291.16)	NA	NA	NA	NA	NA	NA
Dae Jin Nam	SP (*n* = 42)	79.67 (19.96)	462 (186.52)	13.67 (8.44)	NA	83.07 (17.65)	9.93 (6.14)	NA	106.67 (26.87)	5.67 (6.14)
	DP (*n* = 40)	108.33 (19.22)	537 (212.22)	13.4 (7.69)	NA	82.3 (21.53)	10.47 (5.38)	NA	110.23 (30.76)	4 (3.08)
Jun-Feng Liu	SP (*n* = 30)	101.67 (11.68)	NA	NA	NA	NA	NA	NA	NA	NA
	DP (*n* = 32)	124.67 (14.74)	NA	NA	NA	NA	NA	NA	NA	NA
Matthew Gregory Bologna	SP (*n* = 13)	NA	NA	13.4 (9.97)	NA	NA	NA	9.5 (3.74)	102.5 (18.69)	NA
	DP (*n* = 8)	NA	NA	6.75 (0.67)	NA	NA	NA	8.05 (3.35)	83.33 (17.78)	NA
Zhibiao Bai	SP (*n* = 48)	145 (24.9)	513 (312.1)	NA	NA	NA	NA	NA	NA	NA
	DP (*n* = 12)	180 (11.9)	814 (150.6)	NA	NA	NA	NA	NA	NA	NA
Rongbin Sun	SP (*n* = 21)	98.8 (16.1)	301.4 (70.4)	25.45 (2.56)	NA	NA	NA	NA	NA	NA
	DP (*n* = 11)	129.5 (22.4)	359.1 (81.2)	23.41 (3)	NA	NA	NA	NA	NA	NA

b											
Study ID	Groups	Union (%)	Non-union (%)	Malunion (%)	Delayed union (%)	Reoperation (%)	Overall complications (%)	Mortality (%)	Deep SSI (%)	Superficial SSI (%)	Knee joint stiffness (%)
Incheol Kook	SP (*n* = 50)	84	16	NA	NA	16	22	NA	NA	NA	4
	DP (*n* = 27)	100	0	NA	NA	7.41	14.81	NA	NA	NA	7.41
Makoa Mau	SP (*n* = 229)	NA	NA	NA	NA	22.71	NA	2.18	NA	NA	NA
	DP (*n* = 70)	NA	NA	NA	NA	25.71	14.81	4.29	NA	NA	NA
Jerrod Steimle	SP (*n* = 91)	73.63	26.37	NA	NA	NA	14.29	NA	NA	NA	NA
	DP (*n* = 5)	100	0	NA	NA	NA	20	NA	NA	NA	NA
Tyler Thorne	SP (*n* = 133)	92.48	NA	NA	NA	12.78	NA	8.27	5.26	NA	NA
	DP (*n* = 37)	94.59	NA	NA	NA	24.32	NA	13.51	8.11	NA	NA
P. Kriechling	SP (*n* = 66)	NA	13.64	NA	NA	18.18	NA	24.24	NA	NA	0
	DP (*n* = 15)	NA	0	NA	NA	6.67	NA	20	NA	NA	0
Murat Çalbıyık	SP (*n* = 29)	NA	17.24	NA	NA	NA	NA	NA	NA	6.90	NA
	DP (*n* = 27)	NA	7.41	NA	NA	NA	NA	NA	NA	3.70	NA
Nicholas A. Andring	SP (*n* = 34)	NA	5.88	11.76	NA	5.88	NA	NA	NA	NA	NA
	DP (*n* = 38)	NA	0	0	NA	5.26	NA	NA	NA	NA	NA
Chang Heng Liu	SP (*n* = 15)	NA	40	13.33	NA	NA	NA	NA	0	0	0
	DP (*n* = 39)	NA	20.51	2.56	NA	NA	NA	NA	0	2.56	17.95
Dae Jin Nam	SP (*n* = 42)	NA	0	NA	NA	NA	21.43	NA	NA	NA	NA
	DP (*n* = 40)	NA	2.5	NA	NA	NA	7.50	NA	NA	NA	NA
Jun-Feng Liu	SP (*n* = 30)	56.67	13.33	13.33	30	13.33	NA	NA	NA	NA	NA
	DP (*n* = 32)	93.75	0	0	6.25	0	NA	NA	NA	NA	NA
Matthew Gregory Bologna	SP (*n* = 13)	30.77	46.15	NA	23.08	30.77	NA	NA	NA	NA	NA
	DP (*n* = 8)	100	0	NA	0	0	NA	NA	NA	NA	NA
Zhibiao Bai	SP (*n* = 48)	97.92	2.08	NA	NA	NA	NA	NA	NA	NA	NA
	DP (*n* = 12)	100	0	NA	NA	NA	NA	NA	NA	NA	NA
Rongbin Sun	SP (*n* = 21)	NA	9.52	NA	NA	NA	NA	NA	NA	NA	NA
	DP (*n* = 11)	NA	0	NA	NA	NA	NA	NA	NA	NA	NA

DP: Dual Plate, NA: Not Available, ROM: Range of Motion, RUST: Radiographic Union Scale in Tibia, SP: Single Plate

All data presented as mean (SD)

DP: Dual Plate, NA: Not Available, SP: Single Plate, SSI: Surgical Site Infection

A systematic review and meta-analysis of 13 Retrospective studies with an average study duration of 7.77 years (ranging from 4 to 14 years). The analysis encompassed a total of 1015 patients with distal femoral fractures, of whom 654 received single locking plate technique, while 361 received dual plate technique (Table 2).

**Table 2:** Included studies characteristics

Study ID	Year	Country	Duration of study	Type of study	All cohort Sample size	Study arms	*N*	Age, mean (SD)	BMI, mean (SD)	Sex *N* (%)	
										Male	Female
Incheol Kook	2025	Korea	10 years	Retrospective study	77	SP	50	60.4 (19.3)	24.9 (4.1)	30 (60)	20 (40)
						DP	27	60.9 (20.7)	23.4 (4.3)	17 (63)	10 (37)
Makoa Mau	2025	USA	6 years	Retrospective study	152	SP	82	74.1 (10.2)	29.6 (8.8)	23 (28)	59 (72)
						DP	70	75.3 (9.2)	30.3 (8.3)	12 (17.1)	58 (82.9)
Jerrod Steimle	2025	USA	4 years	Retrospective study	96	SP	91	61.5 (30.48)	NA	2 (40)	3 (60)
						DP	5	52.8 (30.48)	NA	33 (36.3)	58 (63.7)
Tyler Thorne	2025	USA	10 years	Retrospective study	170	SP	133	63 (17)	NA	42 (32)	91 (68)
						DP	37	64 (20)	NA	7 (19)	30 (81)
P. Kriechling	2024	UK	14 years	Retrospective study	81	SP	66	82.67 (8.34)	NA	8 (12)	58 (88)
						DP	15	79.67 (12.27)	NA	2 (13)	13 (87)
Murat Çalbıyık	2023	Turkey	7 years	Retrospective study	56	SP	29	71 (7.5)	28 (2.4)	12 (41.4)	17 (58.6)
						DP	27	75 (8.7)	29 (1.5)	11 (40.7)	16 (59.3)
Nicholas A. Andring	2023	USA	6 years	Retrospective study	72	SP	34	74.8 (7.3)	33.2 (7.7)	5 (14.7)	29 (85.3)
						DP	38	75.9 (11.3)	29.5 (6.3)	7 (18.5)	31 (81.5)
Chang-Heng Liu	2023	Taiwan	11 years	Retrospective study	54	SP	15	35.4	NA	7 (46.7)	8 (53.3)
						DP	39	44	NA	29 (74.4)	10 (25.6)
Dae Jin Nam	2022	Korea	14 years	Retrospective study	82	SP	4	77.1 (15.35)	NA	18 (42.9)	24 (57.1)
						DP	40	75.6 (16.92)	NA	15 (37.5)	25 (62.5)
Jun-Feng Liu	2021	China	5 years	Retrospective study	62	SP	30	62.9 (17.2)	NA	14 (46.7)	16 (53.3)
						DP	32	61.8 (15.3)	NA	12 (37.5)	20 (62.5)
Matthew Gregory Bologna	2019	USA	3 years	Retrospective study	21	SP	13	58.67 (24.92)	27 (1.8)	NA	NA
						DP	8	66.5 (20.55)	29.5 (1.2)	NA	NA
Zhibiao Bai	2018	China	8 years	Retrospective study	60	SP	48	NA	NA	23 (47.9)	25 (52.1))
						DP	12	NA	NA	6 (50)	6 (50)
Rongbin Sun	2018	China	3 years	Retrospective study	32	SP	21	44 (11.4)	NA	13 (62)	8 (38)
						DP	11	46.8 (10.5)	NA	6 (54.5)	5 (45.5)

DP: Dual Plate, NA: Not Available, SD: Standard Deviation, SP: Single Plate

The average total sample size was 78.08 (ranging from 21 to 170), and for the Single locking plate was 50.31 (ranging from 13 to 133) vs. Dual plate was 27.77 (ranging from 8 to 70).

The baseline characteristics of the included studies are summarized in the (Table 3a, b). In the single-plating group, the average age was 63.8 years, ranging from 35.5 to 82.67 years. The mean percentage of males was 39.53%, with a range from 12% to 62%, while females made up 60.48%, ranging from 38% to 88%.

**Table 3:** Demographics and baseline characteristics of single vs. dual plating

a										
Study ID	Study arms	Follow-up (months), mean (SD)	Smoking, *N* (%)	Diabetes, *N* (%)	Fracture type *N* (%)		Medial comminution, *N* (%)	Fracture type, *N* (%)		Mode of trauma (RTA), *N* (%)
					Open	Closed		Native	Periprosthetic	
Incheol Kook	SP	19 (5.1)	14 (28.0)	15 (30.0)	NA	NA	15 (30.0)	37 (74)	13 (26)	NA
	DP	21.9 (8.6)	4 (14.8)	7 (25.9)	NA	NA	13 (48.1)	19 (70.4)	8 (29.6)	NA
Makoa Mau	SP	11.8 (11.9)	8 (9.8%)	NA	7 (8.5)	75 (91.5)	NA	NA	NA	8 (9.8)
	DP	10 (8.5)	5 (7.1%)	NA	11 (15.7)	59 (84.3)	NA	NA	NA	10 (14.3)
Jerrod Steimle	SP	NA	34 (37.4)	NA	25 (27.5)	66 (72.5)	NA	NA	NA	28 (32.2)
	DP	NA	1 (20)	NA	2 (40)	3 (60)	NA	NA	NA	2 (40)
Tyler Thorne	SP	NA	82 (63)	NA	NA	NA	NA	86 (65)	47 (35)	NA
	DP	NA	25 (69)	NA	NA	NA	NA	24 (65)	13 (35)	NA
P. Kriechling	SP	NA	NA	NA	NA	NA	9 (14)	0 (0)	66 (100)	NA
	DP	NA	NA	4 (27)	NA	NA	2 (13)	0 (0)	15 (100)	NA
Murat Çalbıyık	SP	5.6	13 (44.8)	8 (27.6)	NA	NA	NA	NA	NA	NA
	DP	5.1	12 (44.4)	10 (37.04)	NA	NA	NA	NA	NA	NA
Nicholas A. Andring	SP	18.2 (13.8)	16 (47.1)	14 (41.2)	NA	NA	NA	0 (0)	34 (100)	NA
	DP	19.8 (16.1)	6 (14.6)	6 (14.6)	NA	NA	NA	0 (0)	38 (100)	NA
Chang-Heng Liu	SP	47.9 (26.8)	NA	NA	11	4	NA	NA	NA	NA
	DP	39.4 (21.3)	NA	NA	21	18	NA	NA	NA	NA
Dae Jin Nam	SP	15.9 (6.91)	NA	NA	NA	NA	NA	NA	NA	NA
	DP	17.77 (10.77)	NA	NA	NA	NA	NA	NA	NA	NA
Jun-Feng Liu	SP	NA	7 (23.3)	8 (26.7)	NA	NA	NA	NA	NA	9 (30.0)
	DP	NA	8 (25.0)	9 (28.2)	NA	NA	NA	NA	NA	12 (37.5)
Matthew Gregory Bologna	SP	9.67 (7.48)	5 (38.4)	2 (15.4)	4 (30.8)	9 (69.2)	NA	9	4 (30.8)	NA
	DP	15.33 (9.83)	3 (37.5)	2 (25)	1 (12.5)	7 (87.5)	NA	5	3 (37.5)	NA
Zhibiao Bai	SP	NA	NA	NA	25 (52)	23 (48)	NA	NA	NA	20 (41.7)
	DP	NA	NA	NA	11 (91.7)	1 (8.3)	NA	NA	NA	10 (83.3)
Rongbin Sun	SP	NA	NA	NA	2 (9.5)	19 (90.5)	NA	NA	NA	NA
	DP	NA	NA	NA	1 (9.1)	10 (90.9)	NA	NA	NA	NA

b																
Study ID	Study arms	Su classification *N* (%)			AO/OTA classification *N* (%)									Side *N* (%)		
		Type 1	Type 2	Type 3	33A1	33A2	33A3	33B1	33B2	33B3	33C1	33C2	33C3	Right	Left	Bilateral
Incheol Kook	SP	3	4	6	0	9	5	0	0	0	5	9	9	NA	NA	NA
	DP	1	2	5	0	3	2	0	0	0	2	5	7	NA	NA	NA
Makoa Mau	SP	NA	NA	NA	0	0	40	0	0	5	0	0	25	NA	NA	NA
	DP	NA	NA	NA	0	0	29	0	0	4	0	0	19	NA	NA	NA
Jerrod Steimle	SP	NA	NA	NA	4	0	9	2	0	0	1	25	23	NA	NA	NA
	DP	NA	NA	NA	1	0	0	0	0	0	0	0	3	NA	NA	NA
Tyler Thorne	SP	NA	NA	NA	0	0	29	0	0	11	0	0	46	NA	NA	NA
	DP	NA	NA	NA	0	0	11	0	0	3	0	0	10	NA	NA	NA
P. Kriechling	SP	28 (43)	30 (46)	7 (11)	NA	NA	NA	NA	NA	NA	NA	NA	NA	36	29	1
	DP	3 (20)	5 (33)	7 (47)	NA	NA	NA	NA	NA	NA	NA	NA	NA	9	6	0
Murat Çalbıyık	SP	NA	NA	NA	0	5	4	0	0	0	2	5	4	NA	NA	NA
	DP	NA	NA	NA	0	3	5	0	0	0	3	4	5	NA	NA	NA
Nicholas A. Andring	SP	9	15	10	0	12	22	0	0	0	0	0	0	NA	NA	NA
	DP	0	22	16	0	9	29	0	0	0	0	0	0	NA	NA	NA
Chang-Heng Liu	SP	NA	NA	NA	NA	NA	NA	NA	NA	NA	NA	NA	NA	10	5	NA
	DP	NA	NA	NA	NA	NA	NA	NA	NA	NA	NA	NA	NA	21	18	NA
Dae Jin Nam	SP	NA	NA	NA	0	0	0	0	10	1	9	19	3	NA	NA	NA
	DP	NA	NA	NA	0	0	18	0	0	0	3	12	7	NA	NA	NA
Jun-Feng Liu	SP	NA	NA	NA	0	0	12	0	0	0	0	18	0	NA	NA	NA
	DP	NA	NA	NA	0	0	10	0	0	0	0	14	8	NA	NA	NA
Matthew Gregory Bologna	SP	NA	NA	NA	NA	NA	NA	NA	NA	NA	NA	NA	NA	NA	NA	NA
	DP	NA	NA	NA	NA	NA	NA	NA	NA	NA	NA	NA	NA	NA	NA	NA
Zhibiao Bai	SP	NA	NA	NA	0	0	16	0	0	6	0	0	26	NA	NA	NA
	DP	NA	NA	NA	0	0	0	0	0	0	0	0	12	NA	NA	NA
Rongbin Sun	SP	NA	NA	NA	0	0	13	0	0	0	0	0	8	NA	NA	NA
	DP	NA	NA	NA	0	0	6	0	0	0	0	0	5	NA	NA	NA

DP: Dual Plate, NA: Not Available, SD: Standard Deviation, SP: Single Plate

AO/OTA: Arbeitsgemeinschaft für Osteosynthesefragen / Orthopaedic Trauma Association, DP: Dual Plate, NA: Not Available, SP: Single Plate

The average BMI reported in five studies was 28.54, with a range from 24.9 to 33.2. The follow-up period across seven studies averaged 18.3 months, ranging from 5.6 to 47.9 months (Table 4).

**Table 4:** Quality assessment of the studies

Study ID	Selection (0–4)	Comparability (0–2)	Outcome/Exposure (0–3)	Total Score (0–9)	Quality
Incheol Kook	★★★★	★★	★★★	★★★★★★★★★	High
Makoa Mau	★★★★	★★	★★★	★★★★★★★★★	High
Jerrod Steimle	★★★★	★★	★★★	★★★★★★★★★	High
Tyler Thorne	★★★★	★★	★★★	★★★★★★★★★	High
P. Kriechling	★★★★	★★	★★★	★★★★★★★★★	High
	★★★★	★★	★★★	★★★★★★★★★	High
Murat Çalbıyık	★★★★	★★	★★★	★★★★★★★★★	High
Nicholas A. Andring	★★★★	★★	★★★	★★★★★★★★★	High
Chang Heng Liu	★★★★	★★	★★★	★★★★★★★★★	High
Dae Jin Nam	★★★★	★★	★★★	★★★★★★★★★	High
Jun-Feng Liu	★★★★	★★	★★★	★★★★★★★★★	High
Matthew Gregory Bologna	★★★★	★★	★★★	★★★★★★★★★	High
Zhibiao Bai	★★★★	★★	★★★	★★★★★★★★★	High
Rongbin Sun	★★★★	★★	★★★	★★★★★★★★★	High

The mean percentage of smokers reported in eight studies was 36.48%, with a range from 9.8% to 63%. The percentage of patients with diabetes mellitus across six studies averaged 25.05%, ranging from 9.4% to 41.2%.

Regarding fracture type, the average percentage of open fractures was 33.6% (ranging from 8.5% to 73.3%), while closed fractures accounted for 66.4% (ranging from 26.7% to 91.5%). For fracture type (native or periprosthetic), the mean percentages reported in four studies were 34.75% for native fractures (range 0% to 74%) and 58.36% for periprosthetic fractures (range 26% to 100%).

In the Dual-plating group, the average age was 64.86 years, ranging from 44 to 79.67 years. The percentage of males was 38.46% (range: 13% to 74.4%), and the percentage of females was 61.54% (range: 25.6% to 87%).

The mean BMI reported in five studies was 28.34, with a range from 23.4 to 30.3. The follow-up period reported in seven studies averaged 18.47 months (range: 5.1 to 39.4 months). The percentage of smokers in eight studies was 29.05% (range: 7.1% to 69%).

The percentage of patients with diabetes mellitus reported in six studies was 26.29% (range: 14.6% to 37.04%). The mean percentage of fracture types (open or closed) across six studies was 37.13% for open fractures (range: 12.5% to 91.7%) and 62.87% for closed fractures (range: 8.3% to 90.9%).

Additionally, the mean percentage of fracture types (Native or Periprosthetic) in four studies was 33.85% for Native fractures (range: 0% to 70.4%) and 60.42% for Periprosthetic fractures (range: 29.6% to 100%).

Su classification reported three studies, the mean percentage type1 vs. type 2 vs. type 3 was 25.16 (ranging from 6 to 43) vs. 32.71 (ranging from 8 to 46) vs. 17.47 (ranging from 11 to 29.41) in single-plating group. in contrasting, the mean percentage was 7.9 (ranging from 0 to 20) vs. 32.77 (ranging from 7.41 to 57.89) vs. 35.88 (ranging from 18.52 to 47) in dual plating group.

The other variables (medial comminution, AO/OTA Classification, side (right, left, or bilateral), mode of trauma (RTA)) are presented in the **(**Table 2a, 2b**)**.

**Fig. 2:**
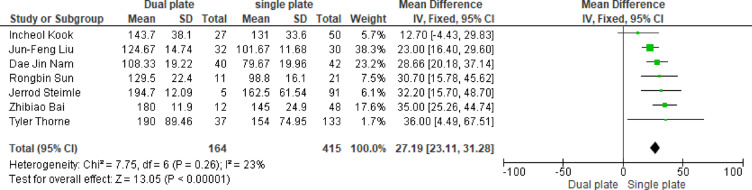
MD of operation time (min)

A meta-analysis of seven studies demonstrated that dual plating was associated with a significantly longer operative time compared to single plating (MD = 27.19 min, 95% CI: 23.11–31.28; *p* < 0.00001). The pooled results were homogenous (*p* = 0.26, I^2^ = 23%) (Fig. 2).

**Fig. 3:**
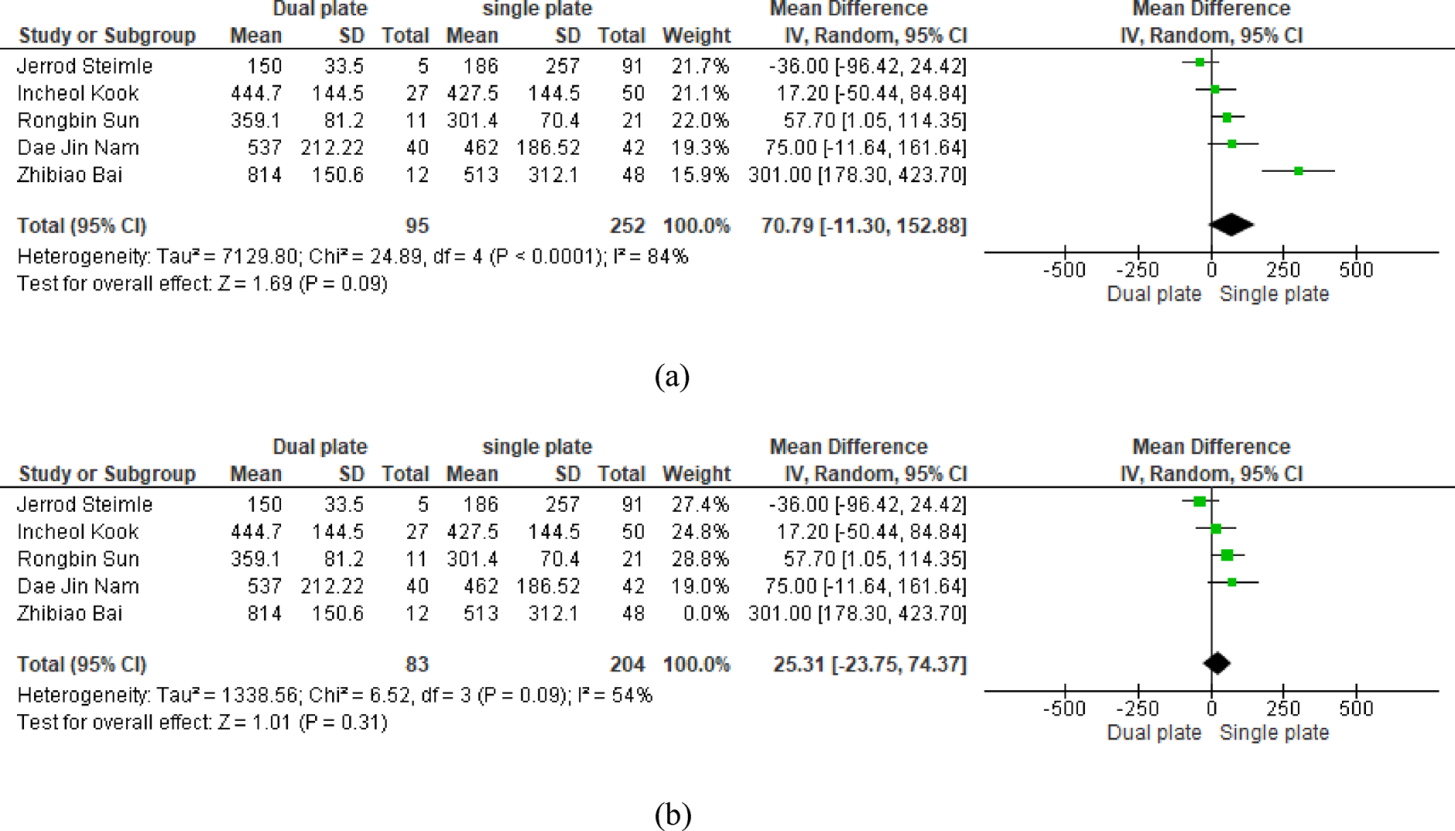
**a** MD of blood loss (ml). **b** MD of blood loss (ml) after doing one-leave test (sensitivity analysis)

Five studies showed an increase in intraoperative blood loss in the dual plating group (MD = 70.79 ml, 95% CI: -11.30, 152.88; *p* = 0.09) without statistical significance. The pooled results were heterogenous (*p* < 0.0001, I^2^ = 84%) and resolved significantly by sensitivity analysis (leaving one study; Zhibiao Bai (*p* = 0.09, I^2^ = 54%) (MD = 25.31 ml, 95% CI: -23.75, 74.37; *p* = 0.31)] (Fig. 3a and b).

The pooled analysis of four studies revealed no significant difference in hospital stay between the two groups (MD = 0.17, 95% CI: -0.39, 0.73; *p* = 0.55). Heterogeneity was absent (*p* = 0.79, I^2^ = 0%), suggesting a high level of consistency in findings across studies (Supplementary file).

**Fig. 4:**
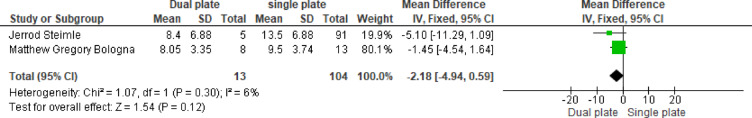
MD of time to full weight bearing (weeks)

Two studies indicated a non-significant trend toward earlier full weight bearing in the dual plating group (MD= -2.18, 95% CI: -4.94, 0.59; *p* = 0.12). No heterogeneity was detected (*p* = 0.3, I^2^ = 6%), supporting the reliability of the pooled estimate (Fig. 4).

**Fig. 5:**
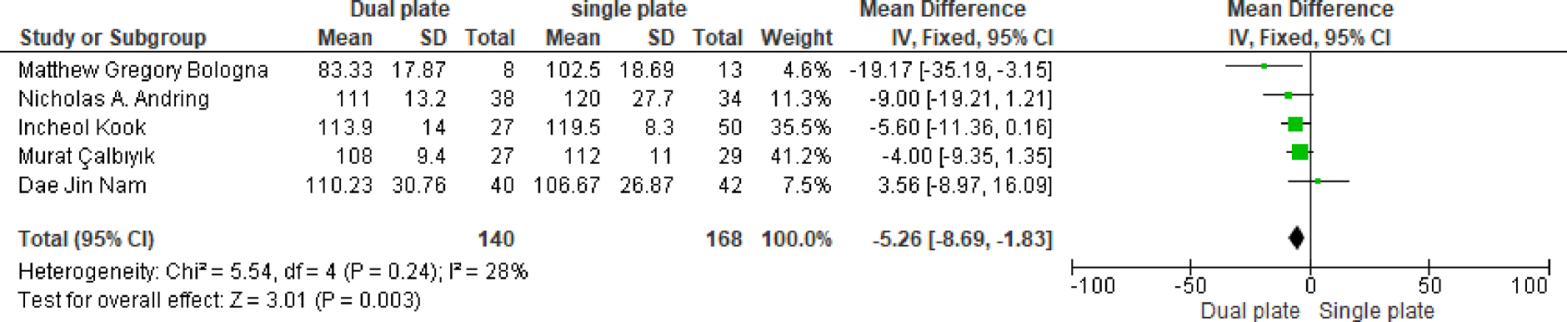
MD of postoperative knee ROM

Analysis of five studies showed that single plating resulted in significantly better postoperative knee ROM (MD= -5.26, 95% CI: -8.69, -1.83; *p* = 0.003). Heterogeneity was low (*p* = 0.24, I^2^ = 28%), indicating mild variability across studies (Fig. 5).

**Fig. 6:**
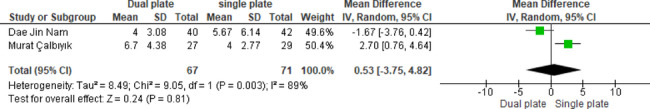
MD of postoperative flexion contracture

Dual plating was associated with insignificant difference, regarding flexion contracture, compared to single plate, based on two studies (MD = 0.53, 95% CI: -3.75, 4.82; *p* = 0.81). Heterogeneity was high (*p* = 0.003, I^2^ = 89%), suggesting variability across studies (Fig. 6).

Two studies showed no significant difference in knee society scores between groups (MD= -0.8, 95% CI: -3.91, 2.32; *p* = 0.62). Heterogeneity was not present (*p* = 0.99, I^2^ = 0%), supporting the uniformity of findings (Supplementary file).

A meta-analysis of two studies showed a non-significant improvement in modified RUST scores in the dual plating group (MD = 0.93, 95% CI: -0.05, 1.91; *p* = 0.06). Heterogeneity was not present (*p* = 0.74, I^2^ = 0%), indicating uniformity in the results (Supplementary file).

Seven studies revealed significantly shorter union time in the dual plating group (MD= -3.08, 95% CI: -5.18, -0.99; *p* = 0.004). Heterogeneity was high (*p* = 0.003, I^2^ = 69%), indicating considerable variability potentially due to differences in fracture types and healing protocols, however; in performing sensitivity analysis, leaving the study by Incheol Kook et al., the heterogeneity was resolved (*p* = 18, I^2^ = 18%). The effect size after the sensitivity analysis also revealed significant difference (MD= -1.86, 95% CI: -3.21, -0.51; *p* = 0.0071) (Supplementary file).

**Fig. 7:**
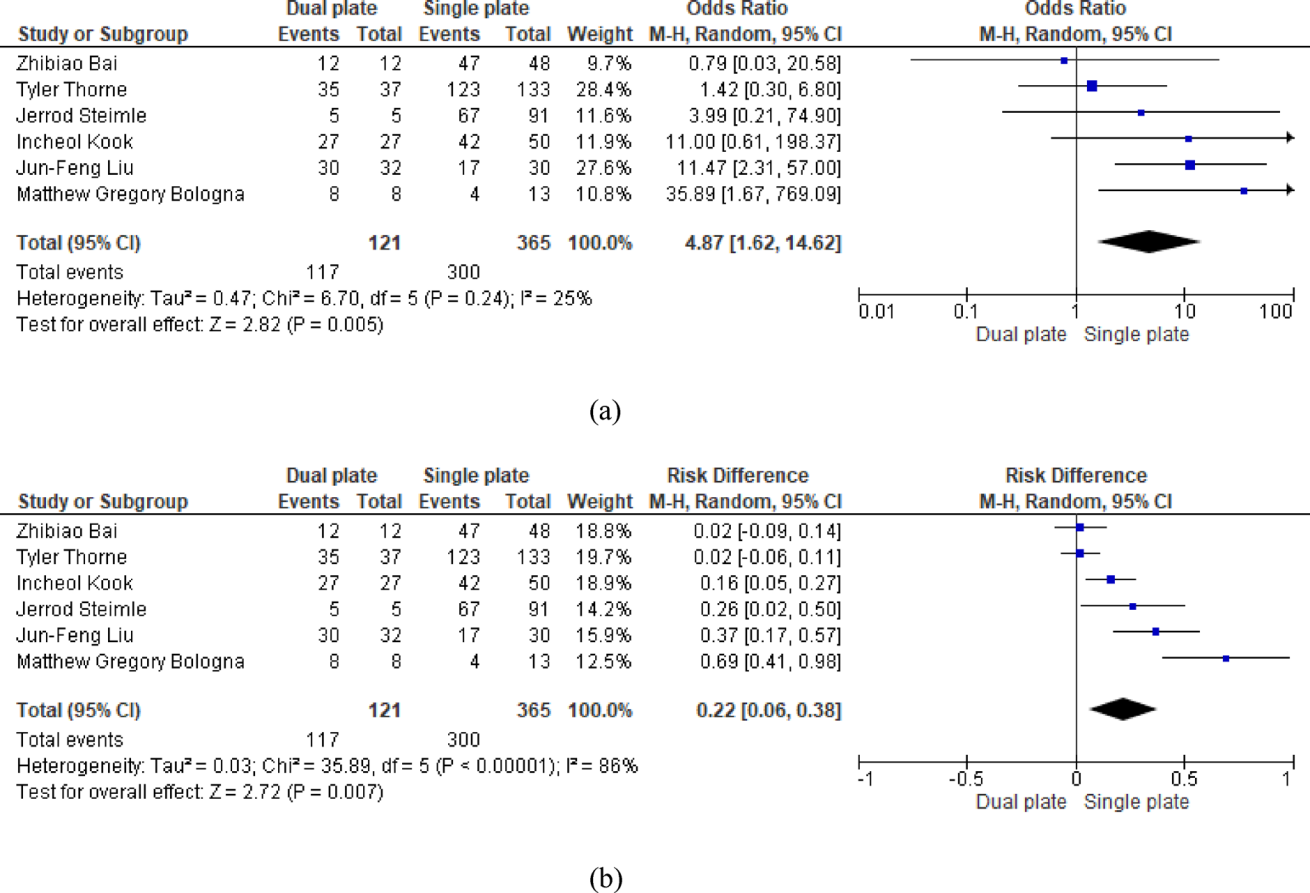
**a** Odds ratio of union rate. **b** Risk difference of union rate

Analysis of six studies showed a significantly higher union rate in dual plate, with odds of union approximately five times greater than single plate (OR = 5.34, 95% CI: 2.23, 12.79; *p* = 0.007). The absolute risk difference showec that treatment with dual plating resulted in 22 additional unions per 100 patients compared with single plating (RD = 0.22, 95% CI: 0.06,0.38; *p* = 0.0002). Heterogeneity was low for the odds ratio (*p* = 0.24, I^2^ = 24.4%), suggesting consistency across studies; however, heterogeneity was high when assessed using risk difference (*p* < 0.00001, I^2^ = 86%) (Fig. 7a and b).

**Fig. 8:**
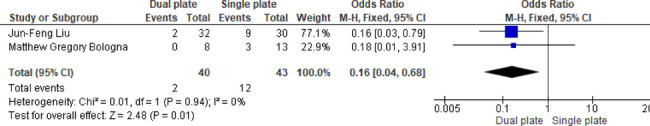
Odds ratio of delayed union rate

Dual plating was associated with an 84% lower odds of delayed union of delayed union across two studies (OR = 0.16, 95% CI: 0.04, 0.68; *p* = 0.01). No heterogeneity was observed (*p* = 0.94, I^2^ = 0%), indicating robust agreement among studies (Fig. 8).

**Fig. 9:**
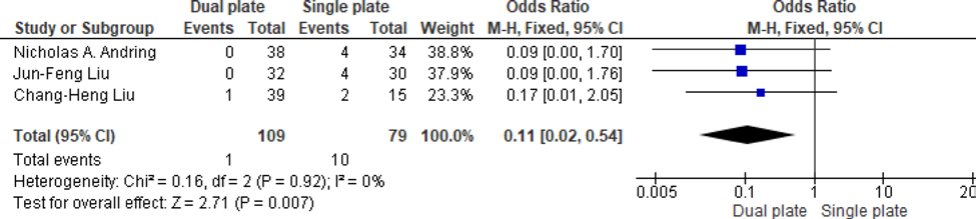
Odds ratio of malunion rate

Three studies reported significantly lower malunion rates in the dual plating group by 89% (OR = 0.11, 95% CI: 0.02, 0.54; *p* = 0.007), with no heterogeneity detected (*p* = 0.92, I^2^ = 0%), enhancing confidence in the findings (Fig. 9).

**Fig. 10:**
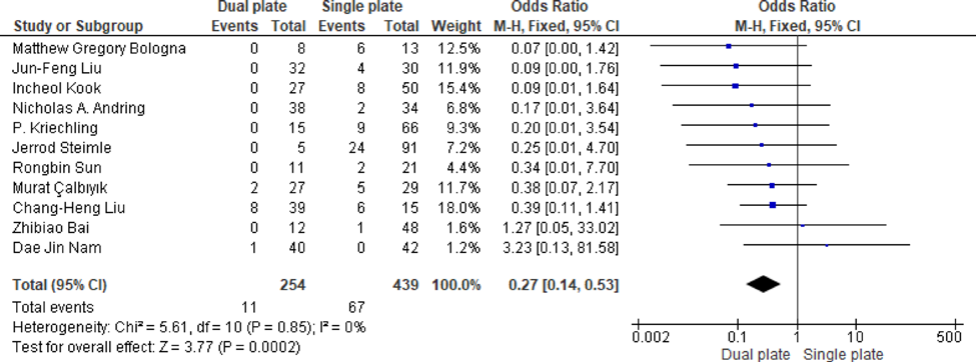
Odds ratio of nonunion rate

**Fig. 11:**
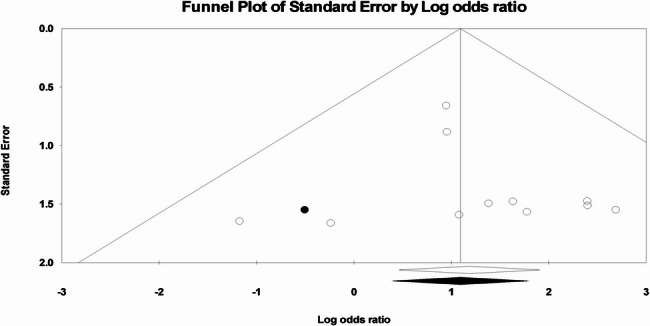
Eager’s regression test for nonunion rate = 0.61105

A total of eleven studies demonstrated significantly lower nonunion rates with dual plating by 73% (OR = 0.27, 95% CI: 0.14, 0.53; *p* = 0.0002). Heterogeneity was absent (*p* = 0.85, I^2^ = 0%), indicating high reproducibility across the included studies (Fig. 10). Funnel plot assessment and Egger’s regression test (*p* = 0.611) indicated no evidence of publication bias (Fig. 11).

**Fig. 12:**
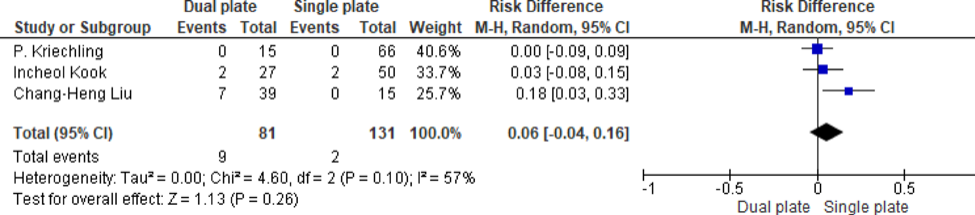
Odds ratio of knee joint stiffness

Three studies reported no significant difference in knee stiffness rates (RD = 0.06, 95% CI: -0.04, 0.16; *p* = 0.26). Heterogeneity was detected (*p* = 0.1, I^2^ = 57%), indicating variability in the results (Fig. 12).

**Fig. 13:**
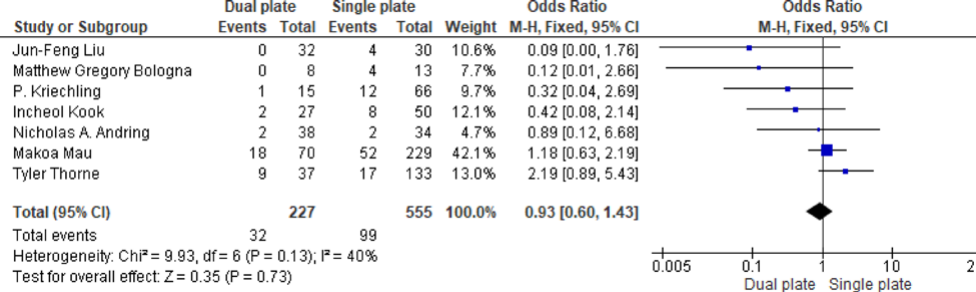
Odds ratio of reoperation rate

Seven studies showed no significant difference in reoperation rates between groups (OR = 0.93, 95% CI: 0.60, 1.43; *p* = 0.73). Moderate heterogeneity was present (*p* = 0.13, I^2^ = 40%), suggesting some variability potentially related to institutional protocols or surgeon preferences (Fig. 13).

Two studies found no significant difference in superficial SSI rates between groups (OR = 0.71, 95% CI: 0.11, 4.65; *p* = 0.72), with no heterogeneity observed (*p* = 0.69, I^2^ = 0%) (Supplementary file).

**Fig. 14:**
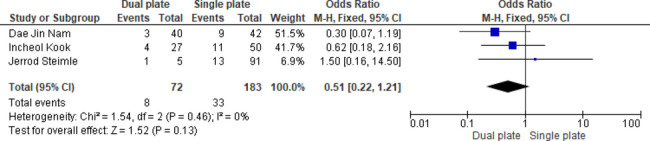
Odds ratio of overall complications rate

Three studies showed no statistically significant difference in overall complication rates between the two groups (OR = 0.51, 95% CI: 0.22, 1.21; *p* = 0.13), and heterogeneity was absent (*p* = 0.46, I^2^ = 0%) (Fig. 14).

## Discussion

The management of distal femoral fractures remains challenging, particularly in cases involving complex fracture patterns or poor bone quality [15]. This systematic review and meta-analysis provides a comprehensive comparison between dual plating and single plating techniques, synthesizing evidence from multiple studies to evaluate their relative effectiveness and safety. While single plating has traditionally been the standard approach, dual plating has gained attention for its potential to enhance mechanical stability and improve healing outcomes [16].

Our meta-analysis reveals that dual plating offers several clinical advantages, particularly in terms of union rates and postoperative knee function, although it is associated with longer operative times, increased intraoperative blood loss, less favorable knee ROM, flexion contracture, and a higher incidence of knee stiffness (although statistically insignificant).

These results have important implications for surgical decision-making and patient-specific treatment planning.

A key distinction was observed in operative parameters. Dual plating was consistently associated with longer surgical duration and, in some studies, increased intraoperative blood loss.

For instance, one study reported an average operation time of 129.5 min for LLP + MAP compared to 98.8 min for LLP (*p* = 0.001) [17], while another showed 183 min for combination fixation versus 134 min for single fixation (*p* < 0.001) [11]. This increase is likely due to the greater technical complexity of dual plating procedures, which typically require additional exposure and soft tissue dissection. Substantial heterogeneity in blood loss (I^2^ = 54%) reflects variability in technique, surgical experience, and patient selection. In contrast, operative time showed low heterogeneity (I^2^ = 23%), indicating more consistent findings across studies.

Despite the increased surgical burden, dual plating did not negatively impact early postoperative outcomes. There was no significant difference in the length of hospital stay, knee stiffness, reoperation rate, superficial surgical site infections, or overall complication rate. These findings suggest that dual plating, while more invasive, does not increase short-term or long-term risk compared to single plating. Furthermore, analysis of publication bias using funnel plots and Egger’s test showed no evidence of bias in nonunion outcomes, supporting the reliability of the results.

While dual plating showed a non-significant trend toward earlier full weight-bearing, this finding is based on a limited number of studies (*n* = 2) and should be interpreted with caution. The scarcity of available data also precluded a reliable subgroup analysis for periprosthetic distal femoral fractures (PDFF), which may have distinct biomechanical and rehabilitation considerations compared to native fractures. Nevertheless, in clinical practice, dual constructs, such as dual plating and plate-nail combinations, are increasingly preferred in PDFF due to their enhanced stability and capacity to support early mobilization [18].This is particularly beneficial in elderly or frail patients, where early weight-bearing improves recovery outcomes such as “Healthy Days at Home” (HDAH 90) and reduces the need for skilled nursing facility care [19]. Notably, the ability of dual constructs to permit immediate weight-bearing challenges the traditional advantage of distal femoral arthroplasty (DFA), making them a viable alternative in select cases [11, 20].

In PDFFs, dual plating has shown promising results, including lower reoperation rates and no revision surgeries in some studies, especially in complex fracture types like comminuted periprosthetic fractures [10].

Biomechanical studies reinforce these clinical findings. Dual constructs provide superior mechanical performance over single plates, including greater axial and torsional stiffness, increased load to failure, and reduced fracture displacement [7]. The addition of a medial plate plays a critical role, particularly when the medial cortical buttress is compromised. It transforms the lateral locking plate’s cantilever configuration into a more stable on-axis construct, improving resistance to varus deforming forces [21–23].

Hybrid configurations combining locking and compression principles on the medial side offer construct rigidity while allowing controlled micro motion at the fracture site, conditions favorable for bone healing [24, 25].

The impact of dual plating on postoperative knee ROM presents a complex and somewhat inconsistent picture across the literature. Although dual plating is often assumed to offer better functional outcomes, our meta-analysis revealed that single plating was associated with significantly better postoperative knee ROM. This finding is supported by several individual studies that reported no significant differences or even favored single plating in terms of ROM outcomes.

For instance, Kook et al. [26] reported comparable mean ROM values across groups, with a numerically higher ROM in the single plating cohort. Notably, one study explicitly found a statistically significantly worse knee ROM for the dual plating group (90.0°) compared to the single plating group (100°) (*p* = 0.036) [27]. Differences may influence these findings in rehabilitation protocols and measurement methods, as many studies relied on clinical estimates rather than standardized goniometer assessments.

Our findings highlight a trade-off between healing outcomes and joint mobility following fixation. Dual plating demonstrated superior healing, with higher union rates and shorter time to union, while single plating was associated with a modest improvement in postoperative knee ROM (MD ≈ 5°, effect size = 0.39). However, this difference in ROM did not translate into improved patient-reported outcomes, as no significant differences were observed in validated functional scores such as KSS. It is important to note that the analysis of KSS was based on only two studies, limiting the strength of conclusions regarding functional outcomes. Taken together, these results suggest that although SP may yield slightly greater ROM, the clinical relevance of this advantage is limited. A gain of approximately 5° is unlikely to meaningfully influence daily function, particularly when balanced against the more consistent and faster union achieved with DP. Therefore, the functional benefit of SP appears modest, and the overall clinical decision should prioritize the more robust healing outcomes observed with DP.

The incidence of flexion contracture appears to be more consistently higher with dual plating. One study showed significantly increased mean flexion contracture (6.7° vs. 4°, *p* = 0.007) [28], while others reported higher rates of stiffness requiring interventions like manipulation under anesthesia [27].

These limitations may be due to increased construct rigidity or more extensive soft tissue trauma. Importantly, these deficits in ROM did not significantly affect broader functional outcome measures. Knee Society Scores and Lysholm scores were comparable between groups across multiple studies, a finding reported by Nam et al. [29].

Nevertheless, flexion contracture may carry particular clinical significance in younger or more active individuals. Even minor losses of terminal extension can alter gait mechanics, impair athletic performance, and predispose to anterior knee pain or early degenerative changes. This highlights the importance of preserving full extension in high-demand patients, where functional consequences are more pronounced [30, 31] .

Dual plating was consistently associated with superior fracture healing outcomes. These included significantly shorter union times, higher union rates, and lower incidences of delayed union, malunion, and nonunion. For example, one study reported a union time of 16.7 weeks with medial-first dual plating versus 22.3 weeks for lateral plating alone [26]; another showed union at 7 weeks with dual constructs compared to 12.5 weeks with single plating [27].

Hybrid medial plating achieved early healing signs in 75% of cases at 3 months, versus 30% with single plating, and resulted in a union rate of 93.8% versus 56.7% [25]. Even in elderly patients with osteoporotic bone, dual plating led to faster healing (13.5 vs. 14.9 weeks) [28]. Some studies documented 100% union rates with dual plating, while single plating was associated with higher rates of nonunion and delayed healing [27, 32].

Although a few studies showed no significant difference, and one even reported longer healing with dual plating, the overall trend across studies supports the mechanical and biological advantage of dual constructs, explaining the observed heterogeneity in union time (I^2^ = 69%).

Delayed union and malunion rates were also markedly lower in dual plating. Delayed union occurred in 0–6% of dual cases versus 23–30% of single plating cases [25, 27]. Malunion or varus collapse was largely absent in dual constructs, compared to up to 26.7% in single plating groups [21, 25]. Nonunion, a critical outcome, consistently favored dual plating. Most studies reported 0% nonunion in dual groups, while single plating groups experienced notable rates [17, 25–27]. Only one study found more nonunion in the dual group (8 vs. 6), but the difference was not statistically significant and was offset by reduced varus collapse [21]. These results collectively support the reliability of dual plating in achieving complete and timely healing across various fracture types.

Finally, while the modified RUST score showed a trend favoring dual constructs, this result was not statistically significant and should be interpreted with caution due to the limited number of studies and potential inter-observer variability in scoring.

This meta-analysis is subject to several limitations. First, most included studies were retrospective, and variability in fixation technique, rehabilitation protocol, and surgical experience could not be fully controlled. Second, the small number of studies for some outcomes (e.g., full weight bearing, flexion contracture, and superficial SSI) limits generalizability. Although our analysis focused on the overall comparison between dual and single plating, it is important to acknowledge that fracture patterns may influence outcomes. Differences between native and periprosthetic fractures, as well as between open and closed injuries, remain underexplored in the available literature. The limited and inconsistent subgroup-specific reporting prevented stratified or meta-regression analyses in the present review. This represents a potential source of residual heterogeneity and should be addressed in future high-quality studies.

## Conclusions

Dual plating offers superior fracture healing outcomes in distal femoral fractures, with union rates approximately five times higher and nonunion risk reduced by nearly 73% compared with single plating, particularly in complex or osteoporotic cases, without increasing complication rates. However, single plating was associated with slightly better postoperative knee range of motion, which may make it a preferable option for selected patients where joint mobility is prioritized. In clinical practice, dual plating may be favored when healing stability is the primary concern, especially in medial comminution or osteoporotic fractures, whereas single plating may be considered in patients where preservation of knee mobility is prioritized.

## Supplementary Information

Below is the link to the electronic supplementary material.

Supplementary Material 1

## Data Availability

All data generated and analyzed throughout this study were included either in this article or its supplementary information file.
