# Supplementary material for: Dual vs. single plating in distal femoral fractures: a systematic review and meta-analysis
Source: J Orthop Surg Res. 2025 Oct 24;20:923. doi: 10.1186/s13018-025-06309-7 (PMC12553283; doi:10.1186/s13018-025-06309-7)
Supplement: Supplementary file 1 — Supplementary Material 1 [file 13018_2025_6309_MOESM1_ESM.docx]

**Dual vs single plating in distal femoral fractures: A Systematic Review and Meta-Analysis**

Ahmed Oun^1*^, Hamdy Khaled Sabra^1^, Omar Abdelaziz^2^, Islam Saeed Elhois^3^, Ahmed Omar Saleh^4^, Khaled Hemdan^5^, Ahmed O. Sabry[^6^](https://ijrsms.com/surgical-audit-of-operated-patients-in-a-tertiary-care-hospital-in-karachi-pakistan/#aff1)

^1^Tanta University Hospital, Faculty of Medicine, Tanta University, Tanta, Egypt.
^2^Faculty of Medicine, Alexandria University, Alexandria, Egypt.
^3^Department of orthopedics, Qena General Hospital, Ministry of health and population, Qena, Egypt.

^4^The University of Jordan, Amman, Jordan.

^5^ October 6 University Hospital, Faculty of Medicine, October 6 University, Giza, Egypt.

^6^Orthopedic department, Cairo University, Cairo, Egypt.

***Corresponding author: Ahmed Oun**

**Tanta University Hospital, Faculty of Medicine, Tanta University, Tanta, Egypt.**

**Email:** [**ahmed_30942176@med.tanta.edu.eg**](mailto:ahmed_30942176@med.tanta.edu.eg)

**Table S1:** Supplementary preferred reporting items for systematic reviews and meta-analyses (PRISMA) checklist.

| **Section/topic** | **Item No** | **Checklist item** | **Reported on page No** |
| --- | --- | --- | --- |
| **Title** | 1 | Identify the report as a systematic review, meta-analysis, or both | 1 |
| **Abstract** | | | |
| **Structured summary** | 2 | Provide a structured summary including, if applicable, background, objectives, data sources, study eligibility criteria, participants, interventions, study appraisal and synthesis methods, results, limitations, conclusions and implications of key findings, systematic review registration number | 2 |
| **Introduction** | | | |
| **Rationale** | 3 | Describe the rationale for the review in the context of what is already known | 3 |
| **Objectives** | 4 | Provide an explicit statement of questions being addressed with reference to participants, interventions, comparisons, outcomes, and study design (PICOS) | 3, 4 |
| **Methods** | | | |
| **Protocol and registration** | 5 | Indicate if a review protocol exists, if and where it can be accessed (such as web address), and, if available, provide registration information including registration number | - |
| **Eligibility criteria** | 6 | Specify study characteristics (such as PICOS, length of follow-up) and report characteristics (such as years considered, language, publication status) used as criteria for eligibility, giving rationale | 4 |
| **Information sources** | 7 | Describe all information sources (such as databases with dates of coverage, contact with study authors to identify additional studies) in the search and date last searched | 4 |
| **Search** | 8 | Present a full electronic search strategy for at least one database, including any limits used, such that it could be repeated | 4 |
| **Study selection** | 9 | State the process for selecting studies (that is, screening, eligibility, included in the systematic review, and, if applicable, included in the meta-analysis) | 4  Fig. 1 |
| **Data collection process** | 10 | Describe the method of data extraction from reports (such as piloted forms, independently, in duplicate) and any processes for obtaining and confirming data from investigators | 4,5 |
| **Data items** | 11 | List and define all variables for which data were sought (such as PICOS, and funding sources) and any assumptions and simplifications made | 4,5 |
| **Risk of bias in individual studies** | 12 | Describe methods used for assessing the risk of bias in individual studies (including specification of whether this was done at the study or outcome level), and how this information is to be used in any data synthesis | 5 |
| **Summary measures** | 13 | State the principal summary measures (such as risk ratio, and difference in means). | 5 |
| **Synthesis of results** | 14 | Describe the methods of handling data and combining results of studies, if done, including measures of consistency (such as I^2^ statistic) for each meta-analysis | 5 |
| **Risk of bias across studies** | 15 | Specify any assessment of risk of bias that may affect the cumulative evidence (such as publication bias, selective reporting within studies) | - |
| **Additional analyses** | 16 | Describe methods of additional analyses (such as sensitivity or subgroup analyses, meta-regression), if done, indicating which were pre-specified | - |
| **Results** | | | |
| **Study selection** | 17 | Give numbers of studies screened, assessed for eligibility, and included in the review, with reasons for exclusions at each stage, ideally with a flow diagram | 6 |
| **Study characteristics** | 18 | For each study, present characteristics for which data were extracted (such as study size, PICOS, follow-up period) and provide the citations | 6  Table 2, 3a, 3b |
| **Risk of bias within studies** | 19 | Present data on the risk of bias of each study and, if available, any outcome-level assessment (see item 12). | 6  Table 4 |
| **Results of individual studies** | 20 | For all outcomes considered (benefits or harms), present for each study (a) simple summary data for each intervention group and (b) effect estimates and confidence intervals, ideally with a forest plot | 8-10 |
| **Synthesis of results** | 21 | Present results of each meta-analysis done, including confidence intervals and measures of consistency | 8-10  Fig. 2-19 |
| **Risk of bias across studies** | 22 | Present results of any assessment of the risk of bias across studies (see item 15) | - |
| **Additional analysis** | 23 | Give results of additional analyses, if done (such as sensitivity or subgroup analyses, meta-regression) (see item 16) | - |
| **Discussion** | | | |
| **Summary of evidence** | 24 | Summarize the main findings including the strength of evidence for each main outcome; consider their relevance to key groups (such as health care providers, users, and policymakers) | 11-13 |
| **Limitations** | 25 | Discuss limitations at the study and outcome level (such as the risk of bias), and at the review level (such as incomplete retrieval of identified research, reporting bias) | 14 |
| **Conclusions** | 26 | Provide a general interpretation of the results in the context of other evidence, and implications for future research | 14 |
| **Funding** | | | |
| **Funding** | 27 | Describe sources of funding for the systematic review and other support (such as the supply of data) and the role of funders for the systematic review | 14 |

**Fig:** SMD of length of hospital stay (days)

**Fig:** SMD of postoperative knee society scores

**Fig:** SMD of modified RUST score (points)

**Fig:** SMD of time to union (weeks)

**Fig:** Odds ratio of superficial SSI rates
